# Supplementary material for: Structures and Anti-Allergic Activities of Natural Products from Marine Organisms
Source: Mar Drugs. 2023 Feb 25;21(3):152. doi: 10.3390/md21030152 (PMC10056057; doi:10.3390/md21030152)
Supplement: Supplementary file 1 [file marinedrugs-21-00152-s001.zip › marinedrugs-2152809-supplementary.pdf]

Supporting information

Structures and Anti-allergic activities of Natural Products from Marine Organisms

Na Chen [1], Shanshan Zhang [1], Ansar Javeed[1], Cuiqin Jian [1], Yi Liu [2], Jinlyu Sun [3], Shandong Wu [2], Peng Fu [4] and Bingnan Han [1,*]

[1] Zhejiang Key Laboratory of Silkworm Bioreactor and Biomedicine, Laboratory of Antiallergic Functional Mole-cules, College of Life Sciences and Medicine, Zhejiang Sci-Tech University, Hangzhou 310018, China.
[2] Hangzhou Zheda Dixun Biological Gene Engineering Co, LTD, Hangzhou, China.
[3] Department of Allergy, Beijing Key Laboratory of Precision Medicine for Diagnosis and Treatment of Allergic Diseases, National Clinical Research Center for Dermatologic and Immunologic diseases, Peking Union Medical College Hospital, Chinese Academy of Medical Sciences, Peking Union Medical College, Beijing 100730, China.
[4] Key Laboratory of Marine Drugs, Ministry of Education of China, School of Medicine and Pharmacy, Ocean University of China, Qingdao 266003, China;
*Corresponding author: Tel: +86-0571-8684-3303.E-mail address: hanbingnan@zstu.edu.cn (B.-N. Han).

Material and Methodology for literature survey:
The review contains original research articles published in English language from 1992 to 2022. During the year of 2022, different database searches are performed on PubMed, Web of Science, MDPI, Elsevier and Springer Link using keywords such as " anti-allergy" + "marine organisms ","anti-allergy" + "mangrove plants ","anti-allergy" + "marine algae ", "anti-allergy + sea corals ", "anti-allergy" + "marine microorganisms ", and combined keyword searches. We only included studies that are completely consistent with the subject of this review. The purpose of this review article is to summarize the recent researches on antiallergic compounds obtained from marine plants, animals and microorganisms, and to provide enough information relevant to the topic of this review article. In order to facilitate readers, we have divided marine organisms into three parts: marine plants, marine animals and marine microorganisms to describe the anti-allergic activity of various compounds produced by them.

Method of molecular docking: To study the interaction between receptors and ligands, molecular docking was performed using Molecular Operating Environment (MOE) software. In MOE, the molecular force field is adjusted to Amber10: EHT, and the Energy Minimize function module is used to minimize the energy of small molecule compounds. The London dG scoring function is employed to create 30 conformations at a time, and the GBVI / WSA dG scoring function is employed. Finally, each result retains 10 conformations, and other parameters are default parameters. Histamine receptor docking model was used for H_1 receptor (pdb: 3RZE).

Table S1: Detailed Information of Marine Natural Products

Compound	Positive control	Name	Structure
1	The stimulated cells with anti-FcεRI antibody	Fucodiphloroethol G	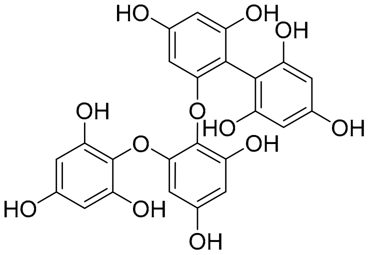
2	The stimulated cells with anti-FcεRI antibody	Eckol	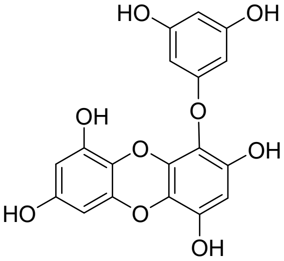
3	The stimulated cells with anti-FcεRI antibody	Phlorofucofuroeckol A	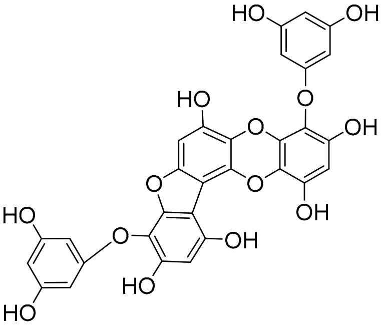
4	A mouse IgG antibody (10 μg/ml)	Dioxinodehydroeckol (DHE)	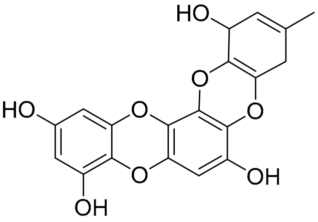
5	A mouse IgG antibody (10 μg/ml)	Phlorofucofuroeckol A (PFF-A)	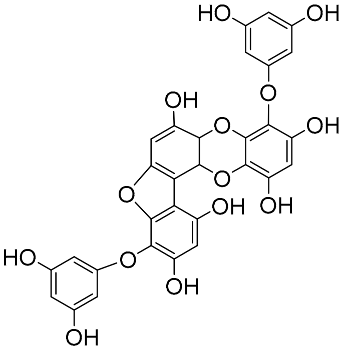

6	UVB-treated cells	Fucofuroeckol-A	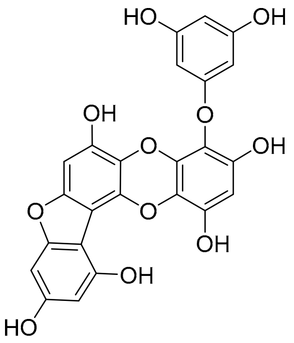
7	With A23187	6,60-bieckol	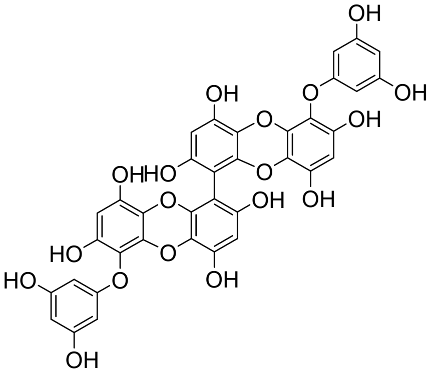
8	With A23187	6,80-bieckol	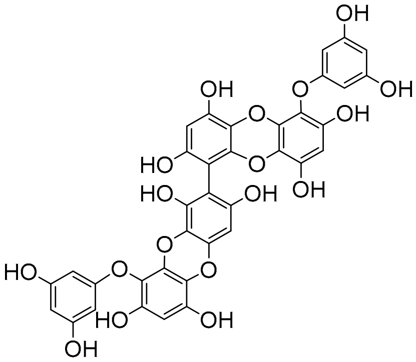
9	With A23187	8,80-bieckol	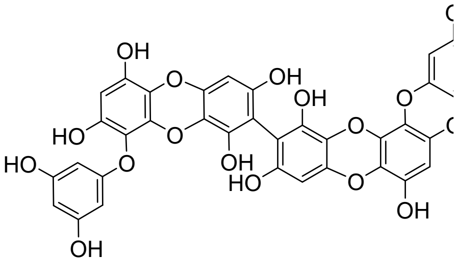
10	With A23187	Phlorofucofuroeckol-B	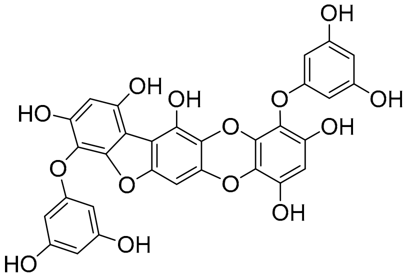

11	Cells treated with DNP-HSA alone	2-[2-(3,5-dihydroxyphenoxy)-3,5-dihydroxyphenoxy]-1,3,5-benzenetriol	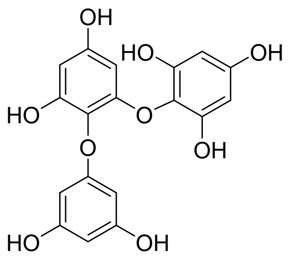
12	Cells treated with DNP-HSA alone	2,2′-[[2-(3,5-dihydroxyphenoxy)-5-hydroxy-1,3-phenylene]bis(oxy)]bis(1,3,5-benzenetriol)	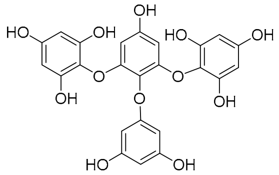
13	Cells treated with DNP-HSA alone	2-[2-[4-[2-(3,5-dihydroxyphenoxy)-3,5-dihydroxyphenoxy]-3,5-dihydrox-yphenoxy]-3,5-dihydroxyphenoxy]-1,3,5-benzenetriol	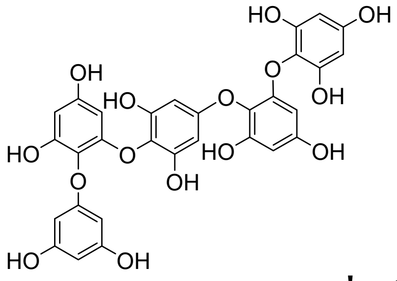
14	Cells sensitized with PCl	Fucoxanthin	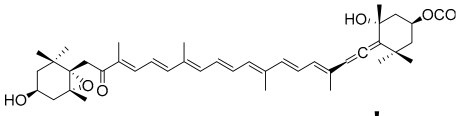
15	Cells sensitized with PCl	Peridinin	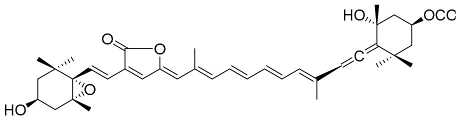
16	Cells received 2% tween-80 water orally	Catechol	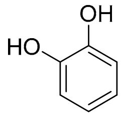
17	Cells received 2% tween-80 water orally	Epicatechin	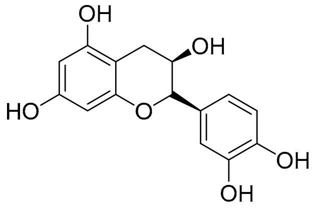
18	Cells received 2% tween-80 water orally	Myricetin	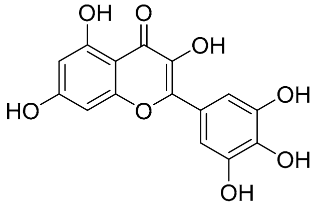
19	Cells received 2% tween-80 water orally	Quercetin	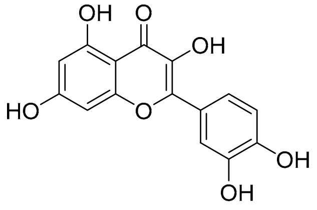
20	Cells received 2% tween-80 water orally	Rosmarinic acid	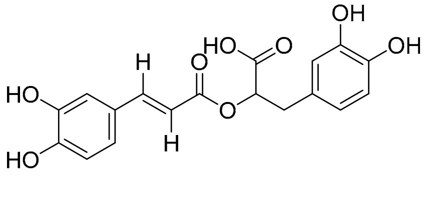

21	Cells received 2% tween-80 water orally	Rutin hydrate	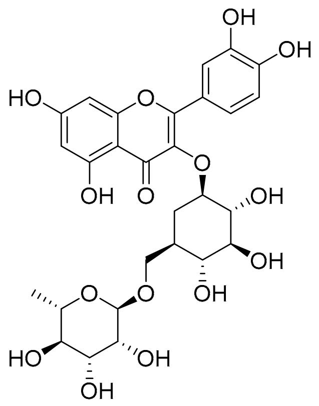
22	Cells received 2% tween-80 water orally	Syringic acid	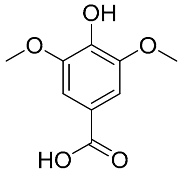
23	Cells received 2% tween-80 water orally	Transferulic acid	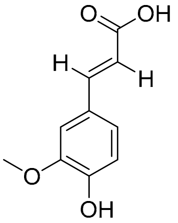
24	Cells received 2% tween-80 water orally	Vanillic acid	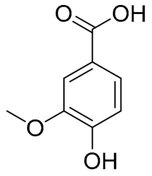
25	DNP-IgE and DNP-HSA unstimulated group	(2-(2-(2-(5-amino-2-(4-amino-2-(3-cyclohexyl-2-(pyrrolidin-1-ium-2-carboxamido)propanamido)-4-oxobutanamido)-5-oxopentanamido)acetamido)-3-hydroxybutanamido)-3-cyclohexylpropanoyl)alanylserinate	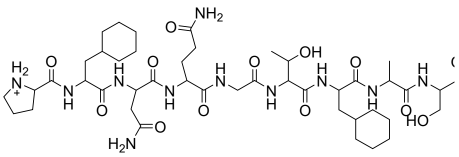
26	Ketotifen	Dysivillosins A	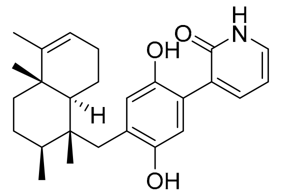
27	Ketotifen	Dysivillosins B	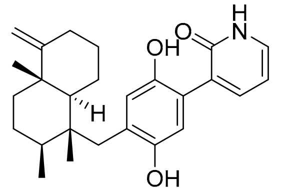

28	Ketotifen	Dysivillosins C	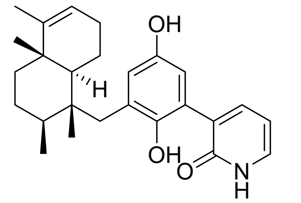
29	Ketotifen	Dysivillosins D	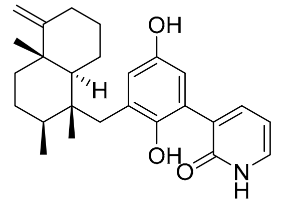
30	Ketotifen fumarate	Hipposponlachnins A	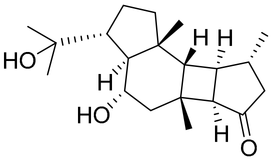
31	Ketotifen fumarate	Hipposponlachnins B	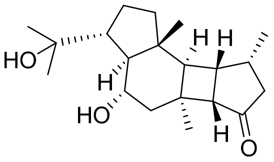
32	Ketotifen fumarate	(1R*,2E,4R*,7E,10S*,11 S*,12R*)-10, 18-diacetoxydolabella -2,7-dien-6-one	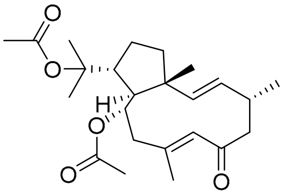
33	Vehicle-treated	IZP-94005	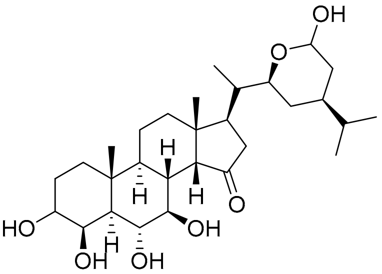
34	Untreated cells	Penasterone	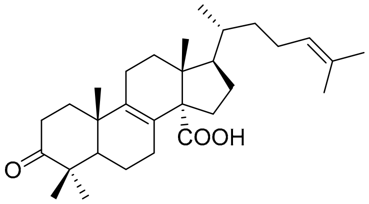
35	Untreated cells	Acetylpenastero	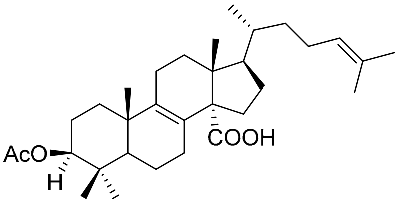
36	Untreated cells	Xestobergsterol A	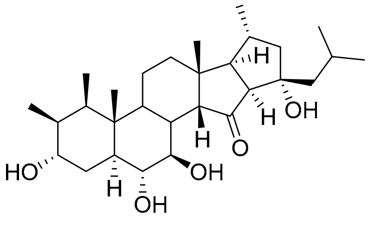

37	Untreated cells	Xestobergsterol B	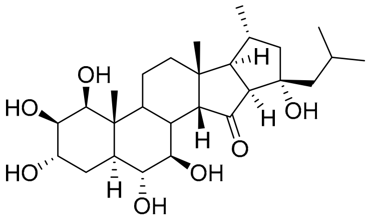
38	Untreated cells	Penastero	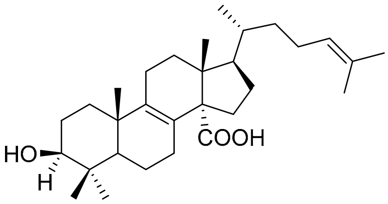
39	Untreated cells	Penasterone	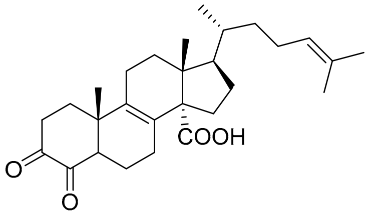
40	Untreated cells	Spinochrome D	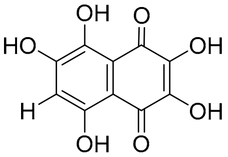
41	Untreated cells	Spinochrome B	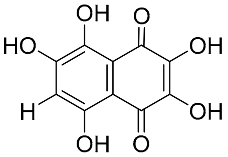
42	Untreated cells	Anhydroethylidene-6,6′-bis(2,3,7-trihydroxy naphthazarin	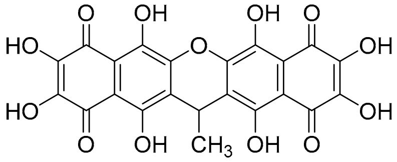
43	Untreated cells	Ethylidene-6,6′bis(2,3,7-trihydroxynaphthazarin	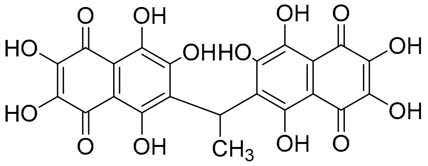
44	Untreated cells	Polyhydroxysteroid	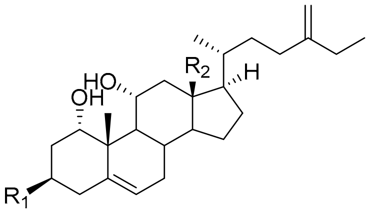
45	Untreated cells	Hyaluromycin	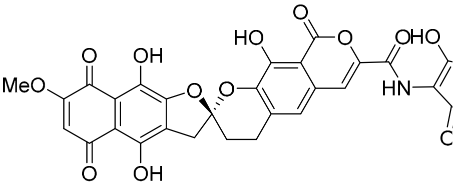
46	Berberine and Loratadine	Reticulol	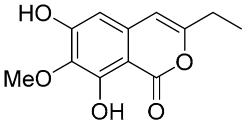

47	Loratadine	Botryotin A	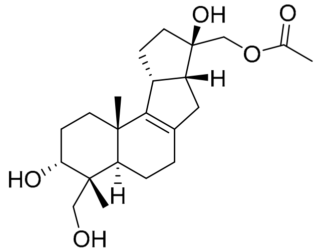
48	The mice in the PBS group were sensitized with 2 mg alum in 200 μL PBS and challenged with PBS	Viridicatol	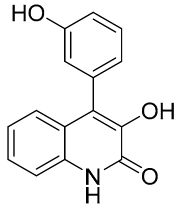
49	Azelastine	Butyrolactones III	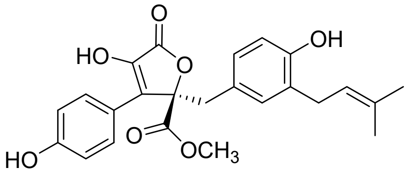
50	Arbutin	α-D-ribofuranoside	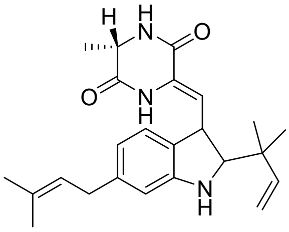
51	Arbutin	3β-(β-D-glucopyranos yloxy)-olean-12-ene-23,28,30-trioic acid	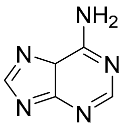
52	Loratadine	Nesterenkoniane	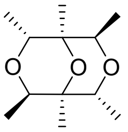
53	Loratadine	Cyclo (D) -proline-(D) -leucine	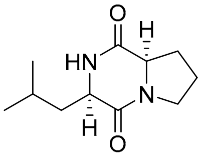
54	Loratadine	Indole-3-carbaldehyd e	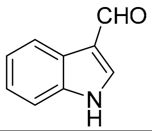
